# Automated classification of RNA 3D motifs and the RNA 3D Motif Atlas

**DOI:** 10.1261/rna.039438.113

**Published:** 2013-10

**Authors:** Anton I. Petrov, Craig L. Zirbel, Neocles B. Leontis

**Affiliations:** 1 Department of Chemistry, Bowling Green State University, Bowling Green, Ohio 43403, USA; 2 Department of Mathematics and Statistics, Bowling Green State University, Bowling Green, Ohio 43403, USA

**Keywords:** RNA 3D motifs, RNA 3D structure, motif classification, FR3D

## Abstract

The analysis of atomic-resolution RNA three-dimensional (3D) structures reveals that many internal and hairpin loops are modular, recurrent, and structured by conserved non-Watson–Crick base pairs. Structurally similar loops define RNA 3D motifs that are conserved in homologous RNA molecules, but can also occur at nonhomologous sites in diverse RNAs, and which often vary in sequence. To further our understanding of RNA motif structure and sequence variability and to provide a useful resource for structure modeling and prediction, we present a new method for automated classification of internal and hairpin loop RNA 3D motifs and a new online database called the RNA 3D Motif Atlas. To classify the motif instances, a representative set of internal and hairpin loops is automatically extracted from a nonredundant list of RNA-containing PDB files. Their structures are compared geometrically, all-against-all, using the FR3D program suite. The loops are clustered into motif groups, taking into account geometric similarity and structural annotations and making allowance for a variable number of bulged bases. The automated procedure that we have implemented identifies all hairpin and internal loop motifs previously described in the literature. All motif instances and motif groups are assigned unique and stable identifiers and are made available in the RNA 3D Motif Atlas (http://rna.bgsu.edu/motifs), which is automatically updated every four weeks. The RNA 3D Motif Atlas provides an interactive user interface for exploring motif diversity and tools for programmatic data access.

## Abstract (precis)

Modular RNA 3D motifs are the building blocks of complex RNA molecules and RNA-based nanomachines, such as the ribosome and the spliceosome. We have created the RNA 3D Motif Atlas, the first comprehensive and regularly updated catalog of RNA hairpin and internal loop motif instances based on a nonredundant set of PDB structures (http://rna.bgsu.edu/motifs). The Motif Atlas provides an interactive user interface for exploring motif diversity and tools for programmatic data access. The potential applications of the Motif Atlas include predicting RNA 3D structures from sequence, designing new RNA nanostructures, and developing new methods for finding RNA 3D motifs in genomic sequences.

## INTRODUCTION

In this paper, we describe a new method for automated classification of internal and hairpin loop RNA three-dimensional (3D) motifs found in RNA structures deposited in PDB, and a new online resource called the RNA 3D Motif Atlas, which presents the results of the motif classification. The RNA 3D Motif Atlas is automatically and regularly updated and can be accessed at http://rna.bgsu.edu/motifs.

### Definitions

In this paper, “RNA 3D motifs” are conceived broadly as “well-defined geometric arrangements of interacting nucleotides.” RNA base pairs and other pairwise interactions meet the definition but are better thought of as submotifs, building blocks of larger motifs. Base triples, quadruples, U- and S-turns, and UA handles are submotifs as well (Jaeger et al. 2009; Abu Almakarem et al. 2012; Agarwal et al. 2012). RNA helices are ubiquitous and well-characterized RNA motifs, and are therefore not the focus of this paper.

An RNA motif is “recurrent” when instances of the same motif are found in nonhomologous locations of the same RNA or different RNAs (Nasalean et al. 2009). Two sets of nucleotides are instances of the “same recurrent motif” when they share the same pattern of interactions and overall geometry. Their sequences need not be identical; there can be base substitutions as well as base insertions or deletions, but the core nucleotides of instances of the same motif should be structurally alignable, nucleotide-by-nucleotide. “Modular” RNA 3D motifs have mutually interacting nucleotides forming an integral unit that can occur in different contexts; many hairpin, internal, and junction loops are modular, and one can often model complex RNA structures by inserting modular 3D motifs into the helical framework defined by the secondary structure. “Local” motifs are composed of nucleotides located close together in the secondary structure and therefore include most hairpin, internal, and junction loops. “Tertiary interaction” motifs involve contacts between nucleotides that are distant in the secondary structure. Examples include pseudoknots, ribose zippers, and loop–receptor interactions. Many modular, recurrent motifs participate in tertiary interactions as one of their functions.

### Purpose of the RNA 3D Motif Atlas

Our motivation for building and maintaining a comprehensive resource focused on RNA 3D motifs stems from the important roles that structured regions of RNA molecules play in living systems, especially in the regulation of gene expression. Numerous new roles for RNA have been discovered, and it is now clear that RNA participates in every phase of gene expression, and not only as a passive carrier of genetic information (mRNA).

A comprehensive collection of recurrent RNA 3D motifs can facilitate the development and evaluation of RNA structure prediction techniques. RNA 3D modeling programs can exploit the modularity of RNA architecture by using the recurrent motifs in the model building process.

The RNA 3D Motif Atlas can also be used to build statistical models for searching for RNA 3D motifs in sequences. This approach is being used to develop JAR3D (CL Zirbel, AI Petrov, J Roll, M Pirrung, NB Leontis, in prep.), a program that calculates the probability of a given sequence to form previously observed, recurrent RNA 3D motifs. The JAR3D web server was used successfully to detect a kink-turn motif in double-glycine riboswitches (Kladwang et al. 2012).

Finally, the knowledge of RNA 3D motif structure and sequence variability can be used to guide experimental studies of RNA 3D motifs. For example, the concept of base-pair isostericity (Stombaugh et al. 2009) has been successfully applied to conduct mutational experiments to investigate several different 3D motifs in the Potato Spindle Tuber Viroid (PSTVd) (Zhong et al. 2007; Takeda et al. 2011).

### Overview of existing motif classification techniques and motif databases

The existing automated motif classification methods can be broadly grouped into two categories, the first of which consists of methods focusing on backbone conformations and their alignments (Wadley and Pyle 2004; Huang et al. 2005; Wang et al. 2007). Although analyses of the phosphate backbone have been successfully used to identify certain motifs, they do not capture base-pairing or stacking interactions, which are often highly conserved and therefore essential for understanding the relationship between RNA sequence and 3D structure. In addition, backbone-oriented methods do not treat variable length insertions or deletions of bulged-out bases even when these do not affect the overall geometry of the core motif.

The second category consists of methods proposed by Djelloul and Denise (2008) and Zhong and Zhang (2012) that use annotations of non-Watson–Crick base pairs to identify and classify RNA 3D motifs. These two methods, however, rely on base-pair annotations and do not incorporate any further geometrical information into their analyses. This limits these approaches when grouping motif instances extracted from poorly modeled 3D structures or distinguishing between closely related motifs having small variations in the annotations of some of their non-Watson–Crick interactions.

In addition to these theoretical limitations, most published methods are limited in the number of motifs or structures analyzed. Furthermore, no previous implementation systematically analyzes new 3D structures as they become available, on an ongoing basis.

The existing manually curated motif databases are either no longer updated (SCOR [Tamura et al. 2004]) or focus only on a limited number of motifs (kink-turn database [Schroeder et al. 2010]). There are also several databases (Andronescu et al. 2008; Bindewald et al. 2008; Popenda et al. 2010; Schudoma et al. 2010a,b; Vanegas et al. 2011) that provide extracted loops for download but do not classify them.

The RNA 3D Motif Atlas aims to combine the best features of the existing methods in an exhaustive and systematic way that provides for continuous updating and archiving.

### Motif classification pipeline

#### Overview of the data processing pipeline

**FIGURE 1.**
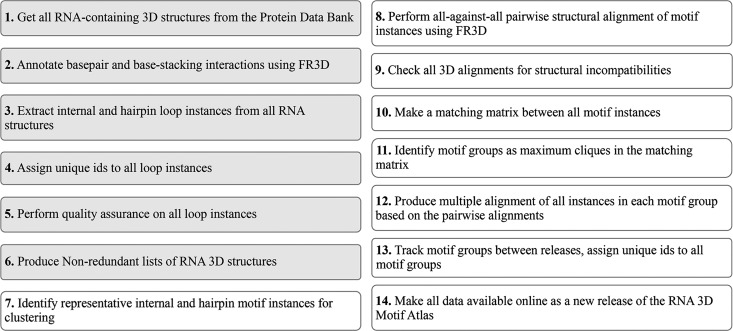
Overview of the data processing pipeline used to produce RNA 3D Motif Atlas releases. Steps 1–6 (gray background) are performed weekly, while Steps 7–14 are performed every 4 wk.

In this section, we describe our approach to motif classification and explain how it is integrated into the automated pipeline for extraction and analysis of internal and hairpin loop RNA 3D motifs (Fig. 1). A weekly automated process downloads all RNA-containing 3D structures from the PDB (Fig. 1, Step 1) and launches the FR3D annotation routines (Sarver et al. 2008) to annotate all pairwise base-pair and base-stacking interactions, as well as “near” interactions (Fig. 1, Step 2).

#### Extracting internal and hairpin loops using FR3D

Each week we extract all hairpin, internal, and junction loops from all RNA-containing 3D structure files (Fig. 1, Step 3) using the FR3D software suite developed and maintained by our group (Sarver et al. 2008).

To facilitate automatic extraction of loop regions from RNA 3D structures, we added a new relation to the FR3D software suite called “flankSS” (“flanks single-stranded region”). It is intended to aid in identifying the nucleotides that form the flanking base pairs that constitute the boundaries of each RNA motif (Hoehndorf et al. 2011). The flankSS relation is motivated by the intuitive concept of “flanking base pairs” or “flanking nucleotides,” which refer to the canonical cWW pairs (GC, CG, AU, UA, GU, or UG) that form the boundaries between RNA hairpin, internal and junction loops, and the Watson–Crick helices to which they are attached. The flankSS relation is a binary, symmetric relationship that is defined to hold between two nucleotides belonging to the same RNA chain if they form canonical cWW pairs (not necessarily with each other) that are nested within the secondary structure of the RNA and if none of the nucleotides between them in the chain forms a canonical nested cWW pair.

The flankSS relation allows one to identify hairpin, internal, and junction loops:

- Hairpin loops are delimited by two nucleotides (nt) that simultaneously make a canonical cWW base pair and satisfy the flankSS relation; this allows the bases in the hairpin to make long-range Watson–Crick pairs called pseudoknots.
- Internal loops are closed by two canonical cWW base pairs provided that on each strand, the nucleotides satisfy the flankSS relation or are adjacent.
- Three-way junctions (3WJ) are closed by three canonical cWW base pairs that bound up to three single-stranded regions.

Details of FR3D searches are included in the Supplemental Material. The method can be extended in a straightforward way to higher-order junctions, but classification of junction motifs is the subject of a future publication and is not discussed here.

The closing bases making canonical cWW pairs are considered to be part of the loop because this helps to better distinguish between different classes of related motifs. It also corresponds to the demarcation of these loops in secondary structures predicted from RNA sequences. And, in some motifs such as the C-loop, the closing base pairs are an integral part of the motif.

#### Assigning unique ids to the loop instances

Once loops are extracted as described above, we label them with unique and stable loop identifiers (ids) (Fig. 1, Step 4). Our intention is to provide unambiguous accession codes for RNA 3D motif instances, which can be used in the future by all workers in the field, for example, by referring to them in publications that can then be found by Internet search engines. To the best of our knowledge, this is the first comprehensive, automated system for assigning identifiers to RNA internal and hairpin loops.

The “loop ids” contain the following three fields, separated by underscore characters:

- Field 1: Loop type prefix: “IL” for internal loops, “HL” for hairpin loops, “J3” for three-way junctions (two characters, all capitals). This can be expanded in the future to larger junctions (“J4,” “J5,” etc.). A similar scheme can be used for tertiary interaction motifs.
- Field 2: PDB id (four characters, all capitals)
- Field 3: A sequentially assigned, three-digit, right-justified, zero-filled integer (starting with “001”). This field does not imply proximity in the sequence, two-dimensional (2D) structure, or in 3D space.

Example loop ids include IL_1S72_001, HL_1J5E_064, and J3_2AVY_001 for an internal, hairpin, and a three-way junction loop, respectively.

#### Loop extraction quality assurance

To ensure the validity of all loops that are included in the Motif Atlas, we carry out the multistage quality assurance process (Fig. 1, Step 5) described in detail in the Supplemental Material. If the 3D position of one or more nucleotides or their constituent parts is not specified in the PDB file, then such a motif instance is set aside and not included in any RNA 3D motif group because its geometry is undetermined and its pairwise interactions cannot be analyzed. At present, motif instances with modified nucleotides are also set aside because FR3D does not annotate pairwise interactions involving nonstandard bases. Currently, ∼9% of all loop instances are excluded from the Motif Atlas based on the quality assurance results (8424 out of 95,624 total internal and hairpin loops from all structures).

#### Selection of loop instances for clustering

A major challenge in working with experimental macromolecular data from the PDB is the large number of very similar structures. For instance, there are many similar structures of the *Escherichia coli* ribosome, and each of these has a large number of motifs. It is desirable to analyze a single representative instance from each motif instead of multiple copies of the same motif from the redundant PDB files. Also, some newer structures are much better modeled than older structures. To work with the best 3D data, our approach is to select a nonredundant set of the best-modeled RNA-containing 3D structures and consider only motifs from those (Fig. 1, Step 6). Selection of a nonredundant (NR) set of RNA-containing 3D structures was described in Leontis and Zirbel (2012). In summary, the entire collection of RNA 3D structures is divided into equivalence classes, and each equivalence class is represented by a single structure chosen according to a set of strictly defined criteria. Note, in particular, that ribosomal structures from different organisms are not considered to be redundant. The procedure is entirely automated and has been running stably every week since February 2011. Improvements in the selection of nonredundant lists will directly impact and improve the RNA 3D Motif Atlas.

Some PDB files have multiple versions of the same chain or chains. To reduce redundancy within PDB files, we keep only loops occurring in the representative RNA chains (Leontis and Zirbel 2012). For example, PDB file 1KOG contains eight chains with the same sequences and almost identical geometries, each with one internal and one hairpin loop. Motif instances from only one of these chains are selected for clustering.

#### Automated motif classification

We select for clustering only RNA loops extracted from the representative structures of the latest 4 Å nonredundant PDB file list that pass all loop quality-assurance steps (Fig. 1, Step 7). These loops are considered to be motif instances and are used as an input for the motif classification. The 4 Å NR list includes only X-ray structures with resolution of 4 Å or better.

**FIGURE 2.**
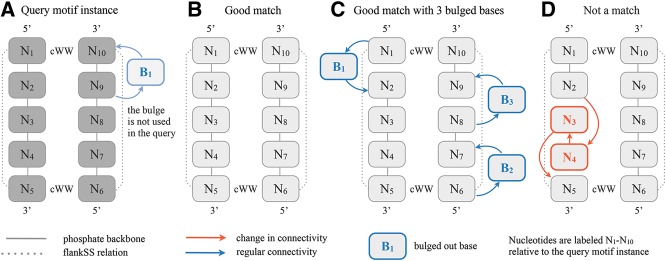
Examples of motif instances that do and do not match a query motif instance in the geometric all-against-all FR3D alignments. (*A*) An 11-nt motif instance. The bulged base B_1_ (transparent blue) is not included in the FR3D query, which only contains 10 nt (dark gray). (*B*) A 10-nt motif instance that matches the query well but does not have the same bulged base. (*C*) A 13-nt motif instance, which has 10 nt that match the query and three bulged bases (highlighted in blue). Due to the omission of the bulged base from the query, the two structures still match despite the four bulged bases in different places. (*D*) This 10-nt motif instance is geometrically similar to the query only when the nucleotides are aligned out of sequence order. The nucleotides are labeled N_1_–N_10_ throughout the figure to indicate which nucleotides are aligned.

The motif instances are compared with each other to identify motif groups. First, we exhaustively align all motif instances with each other using the geometric search capabilities of FR3D. Those that align well enough are said to match (Fig. 2A–C). Next, we identify structurally incompatible motif pairs according to predefined criteria (see below) and remove links between them. Finally, we cluster all motif instances into motif groups by identifying maximum cliques in the graph representing matches between motif instances.

#### All-against-all pairwise structural alignments

The FR3D geometrical search was designed to search RNA 3D structures for all instances resembling a query RNA fragment, up to a specified maximum geometric discrepancy (Sarver et al. 2008). It returns a list of nucleotide-to-nucleotide alignments of the query fragment to each candidate found. We use a FR3D geometrical search to perform all-against-all structural alignments (Fig. 1, Step 8) within the set of loops obtained in the previous step. The alignments are performed using the relatively high geometrical discrepancy cutoff of 1.0 Å per nucleotide, to allow for structural variability. Similar loops belonging to the same motif usually have much lower geometrical discrepancies (0.1–0.6 Å/nucleotide), but some instances that should be considered homologous by their locations at equivalent sites in homologous molecules have higher discrepancy, so we use this less stringent cutoff of 1.0.

To speed up the alignments, three symbolic constraints are imposed on the FR3D searches: (1) The closing pairs are required to make cWW pairs (“near” base pairs defined by FR3D are not allowed). (2) If the flankSS relation holds between two nucleotides in the query structure, then it must also hold between the corresponding nucleotides in the matching structure. (3) Alignments must respect sequential order (5′ to 3′) on each strand (Fig. 2D). These constraints are consistent with the definition of internal and hairpin loops and do not fundamentally restrict the search space. No nucleotide identity or chain length constraints are imposed.

A key difficulty arises when the query motif instance has “bulged” bases that may not be present in all other instances. Motifs that lack the bulged bases will not be found if the bulged bases are included in the search. Therefore, we wish to search with the “core” of the motif and not be distracted by the “bulged” bases. To this end, only nucleotides that make base-pairing or base-stacking interactions with other nucleotides in the motif instance are included when a motif instance is being used as a query, so that it can be found within other instances of the motif. The bulged bases are excluded from queries but not from target motif instances.

To compare all loops extracted from the NR data set with each other, one must carry out more than 2.5 million loop superpositions. To reduce execution time, for internal loops, a preliminary screen is applied before the full structural comparison described above is attempted. The preliminary screen is a structural alignment that determines how well the four closing nucleotides of the query structure superpose onto the four closing nucleotides of the target structure at the same geometric discrepancy cutoff that is used for the full alignment (1.0 Å/nucleotide). Only target loops meeting this criterion are subjected to full alignment. This alignment is very fast and avoids unnecessary and more costly alignments using the entire motif instance.

The output of the all-against-all comparison procedure is a set of nucleotide-level structural alignments for each pair of geometrically similar motif instances. We also record the geometric discrepancy associated with each alignment.

#### Structural incompatibilities between motif instances

Many motif instances that can be aligned by FR3D at geometric discrepancy ≤1.0 Å/nucleotide have incompatible structural features and therefore should not be placed into the same motif group. Several structural criteria are used for detecting such aligned but structurally incompatible matches (Fig. 1, Step 9). These criteria were derived from manual inspection of pairwise alignment results and provide a way of combining the power and inclusiveness of geometric search with knowledge-based criteria often applied in symbolic motif searches.

**FIGURE 3.**
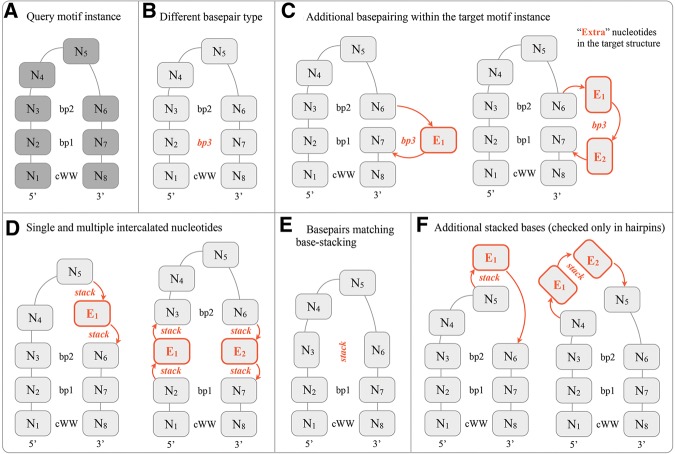
Structural features that cause motif instances aligned during geometric FR3D all-against-all alignments to be marked as structurally incompatible. (*A*) A query 8-nt hairpin loop with a closing canonical *cis*-Watson–Crick/Watson–Crick base pair (cWW) and two noncanonical base pairs labeled *bp1* and *bp2*. Throughout the figure, the nucleotides are labeled N_1_–N_8_ to indicate which nucleotides were aligned with the query. (*B*) Motif instances in the same motif group must not have base pairs from different base-pair families. In this case, *bp1* is from a different family than *bp3*. (*C*) The query is aligned to a larger structure that has additional “extra” nucleotides (highlighted in orange). If the extra nucleotides are involved in base-pairing interactions with other nucleotides forming the loop, then such motif instances are structurally incompatible. (*D*) Motif instances are also incompatible when extra nucleotides intercalate between aligned nucleotides. (*E*) When nucleotides making base pairs are aligned with nucleotides making stacking interactions, such motif instances are incompatible. (*F*) In hairpins an additional criterion is used. Motif instances in the same family must not have unaligned nucleotides stacked on the aligned ones or on each other.

First, if aligned nucleotides in the query and in the target structure form base pairs belonging to different geometrical families according to the Leontis–Westhof classification (Leontis and Westhof 2001), then such motif instances are marked as being structurally incompatible, which will result in them being assigned to different motif groups (Fig. 3A,B). This criterion is based on the idea that motifs that have base pairs belonging to different geometric families at equivalent positions will likely exhibit different sequence variation signatures and should be considered distinct (Klostermeier and Hammann 2013).

Although the query and the target loops need not be the same size, the nucleotide-level alignments generated by FR3D necessarily have the same number of aligned positions as the query. As a result, an 8-nt loop can “match” a 10-nt loop, leaving two “extra” nucleotides in the target motif instance unaligned to the query. This allows a motif group to include instances with variable numbers of bulged bases that do not otherwise change the structure of the motif. However, one has to check the “extra” nucleotides carefully in the target structure to make sure that they are, indeed, bulged out and that the overall architecture of the target loop resembles that of the query loop. This leads to two more criteria: The “extra” bases should not make any “true” or “near” base pairs, as annotated by FR3D, with the other nucleotides of the loop (Fig. 3C), and the bases of “extra” nucleotides should not intercalate between other bases of the loop (Fig. 3D). An “extra” nucleotide is determined to intercalate if it is involved in two or more stacking interactions with other bases of the loop.

The next criterion addresses the case in which the aligned bases base-pair in the first structure but stack in the second. When this occurs, the two structures are sufficiently different to be assigned to different motif groups (Fig. 3E). In some cases, we find that this indicates that one or both structures are poorly modeled. Filtering out such initial matches produces more coherent motif groups.

The criteria discussed so far are applied to both internal and hairpin loops. For hairpins, we use an additional structural criterion to deal with the fact that many nucleotides forming hairpins interact with other nucleotides in the same loop, only via stacking interactions. When one or more “extra” nucleotides stack on top of the aligned bases or when several “extra” nucleotides stack on each other, the motif instances are marked as structurally incompatible (Fig. 3F).

Structurally incompatible motif instances will be put into different motif groups, but their geometric discrepancy is saved and is used to identify structurally related motif groups, because two motifs may have distinct base-pairing patterns and yet adopt a similar shape in 3D space. Both pieces of information are useful because the base-pairing pattern dictates the sequence variability of the motifs, while the overall 3D similarity can be used to identify motifs that can substitute for each other (“motif swap”) (Nasalean et al. 2009).

All structural incompatibility annotations are stored in the database and are presented online using a special interface when one compares two motif groups to ascertain incompatibilities between their motif instances.

#### Matching matrix and maximum cliques

We organize the alignment and incompatibility results into a square matrix, called the “matching matrix” (Fig. 1, Step 10), where the rows represent motif instances when used as search queries, the columns represent instances when used as targets, and the matrix elements contain alignment and incompatibility information. The diagonal cells of the matching matrix are set to zero because the geometric discrepancy of any structure with itself is zero. Cells (*i*, *j*) and (*j*, *i*) correspond to two different FR3D searches. If neither search results in a match, both entries are set to infinity. If either search gives a structurally incompatible match, both the (*i*, *j*) and (*j*, *i*) entries are set to infinity. Otherwise, both the (*i*, *j*) and the (*j*, *i*) entries are set to the lowest geometric discrepancy among the two searches. The matching matrix is thus symmetric.

We adopt a graph-theoretical approach to identify motif groups. The finite entries of the matching matrix define a graph, where each motif instance is represented by a node of the graph and is connected by a weighted edge to every other instance that it matches. In this scheme, a “motif” is a cluster of pairwise geometrically similar and compatible motif instances and therefore corresponds to a subgraph of maximally connected nodes. Such subgraphs in graph theory are known as “cliques.” Thus, finding the motif groups using the matching matrix is equivalent to finding the largest cliques (or “maximum cliques”) in the corresponding graph.

We use the exact maximum clique–finding algorithm implemented in R3D Align (Rahrig et al. 2010) to iteratively find the largest clique, remove it from the graph, and continue to the next largest clique using the remaining instances. If at any stage there are two or more maximum cliques of the same size, we favor the one with the lowest sum of geometric discrepancies (the more tightly connected clique). This procedure ensures that the clique extraction procedure is reproducible regardless of the ordering of the instances in the matching matrix (Fig. 1, Step 11).

Following the procedure described above, we obtain a list of motif groups and their motif instances. The last step is to construct multiple structural alignments (Fig. 1, Step 12) for instances in each motif group from the pairwise nucleotide level alignments computed between the instances during the exhaustive pairwise comparison stage. This is done by identifying the nucleotides that are aligned in all instances, which always include the flanking bases because of the constraints imposed on FR3D searches. Any additional nucleotides that are aligned in the pairwise alignments between the members of the clique are added to the consensus multiple alignment. This consensus defines the “core nucleotides” of the motif group. For example, an 8-nt internal loop instance could be aligned with a 6-nt and a 7-nt loop. Their consensus alignment may contain 4–6 nt depending on the number of bulged-out or unaligned bases. The aligned motif instances are the final product of motif classification and become part of a Motif Atlas release.

### Motif Atlas releases

Every 4 wk new internal and hairpin loop Motif Atlas releases are made available, and each release is assigned a “release id” consisting of two integers separated by a dot. The first number conveys change significance and is incremented when the programs undergo significant modifications. The second number is assigned consecutively to each release starting at 1. For example, the 1.0 internal loop release is the first official release, while the 0.6 internal loop release is a preliminary Motif Atlas release. Internal and hairpin loop motifs have separate release ids because it may be necessary to update them asynchronously.

### Unique and stable ids for motif groups

We assign identifiers (ids) to each motif group. This helps track motifs and their instances between releases and facilitates data archiving (Fig. 1, Step 13). “Motif group ids” consist of three fields, with the first two fields separated by underscores and the last field separated by a dot:

- Field 1: Loop type prefix (“IL” for internal loops, “HL” for hairpin loops)
- Field 2: Five-digit unique randomly assigned integer
- Field 3: Version number (an integer starting at 1; see below)

Examples of motif ids include IL_24982.1 and HL_94618.2. This scheme can be extended to naming junction motifs by using J3, J4, etc. as loop type prefixes. Motif ids are distinct from the loop ids described above. Loop ids label individual hairpin or internal loop motif instances, while motif ids refer to motif groups, which are collections of motif instances.

### Tracking motif groups between releases

It is helpful to track the motif groups with each release to distinguish new motifs from those that have been updated by adding, removing, or replacing some motif instances. The use of a “version number” in motif group ids allows us to maintain the same base id for stable motif groups, while also recording when changes occur between releases. For example, the addition of new triple-sheared motif instances to the triple-sheared motif group need not result in the creation of a brand-new id for the triple-sheared motif, because it already contains a large number of members, so only the version number will be incremented (for example, motif id IL_87904.1 has been incremented to IL_87904.2 and then to IL_87904.3). Release 1.0 contains several motif groups with version numbers as high as 5, indicating that new motif instances were added four times to an otherwise stable motif group.

**FIGURE 4.**
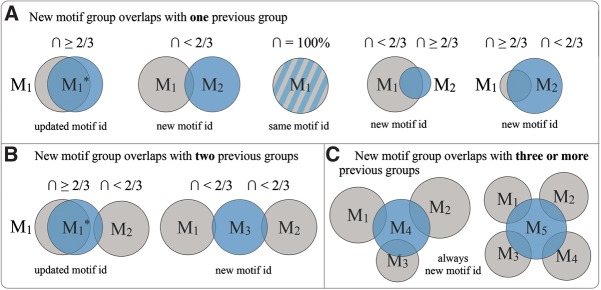
Motif group id assignment in successive Motif Atlas releases. Motif groups are shown as blue (new release) and gray (previous release) circles with ids M_1_, M_2_, M_3_, etc. New motif groups are shown overlapping with (*A*) one previous group, (*B*) two previous groups, and (*C*) three or more previous groups. (*) Updated motif ids. The case in which the identical motif group is present in the new and previous releases is indicated by blue and gray stripes. The sizes of the circles indicate the number of instances in the motif groups.

In successive releases of the Motif Atlas, motif group ids are assigned according to the following rules:

- If all instances of an already existing motif group exactly match a motif group in the next release of the Motif Atlas, then the same id is used in the new release without incrementing the version number (Fig. 4A, top center).
- If a motif group in the new release shares motif instances with one or two motif groups of the previous release, then an attempt is made to identify a match where the number of shared motif instances is greater than or equal to two-thirds of the sizes of both the new and old motif groups. In this case, the successor motif group in the new release is assigned the same id as the motif group in the previous release, but its version is incremented. If the overlap between the motif groups from the new and previous releases is less than two-thirds of the sizes of either group, then a new id is created (Fig. 4A,B).
- If a motif group in the new release has common motif instances with more than two motif groups in the previous release, then a new id is created regardless of the number of common instances (Fig. 4C).
- If a motif group in the new release does not share any motif instances with any motif group in the previous release, then a new id is automatically generated.

The two-thirds cutoff is reasonable for both large and small motif sizes, and manual inspection of motif histories across releases indicates that it works well given the current rate at which new 3D structures are deposited in PDB. As the RNA 3D Motif Atlas evolves, this cutoff may be adjusted as necessary.

### Annotating and curating the Motif Atlas

Some recurrent RNA 3D motifs have established names and synonyms (for example, the sarcin–ricin motif is also known as the G-bulge or S-motif). Many Motif Atlas entries have been manually annotated with commonly used motif names and information regarding their locations in ribosomal RNA (large and small subunit helix numbers). These manually added annotations are associated with motif ids and are automatically propagated to the successor motif ids in subsequent Motif Atlas releases. Following the success of databases like Rfam in allowing the research community to create annotations using wikis, a similar strategy can be adopted in the future versions of the Motif Atlas.

## RESULTS

### RNA 3D Motif Atlas Release 1.0

The first official release of the RNA 3D Motif Atlas (internal and hairpin loop releases 1.0) occurred on March 4, 2013. It contains 267 internal loop motif groups with 1524 total motif instances and 252 hairpin loop motif groups with 991 motif instances. It is based on the 4 Å nonredundant list version 1.0 containing 710 RNA 3D structures (http://rna.bgsu.edu/rna3dhub/nrlist).

### Sarcin–ricin, kink-turn, C-loop, GNRA, T-loop, UNCG motifs

We have carried out extensive manual evaluation of the motif classification results, which demonstrated that known RNA 3D motifs such as sarcin–ricin, kink-turn, C-loop, T-loop, GNRA, and UNCG loops were successfully identified and placed into homogeneous groups.

**FIGURE 5.**
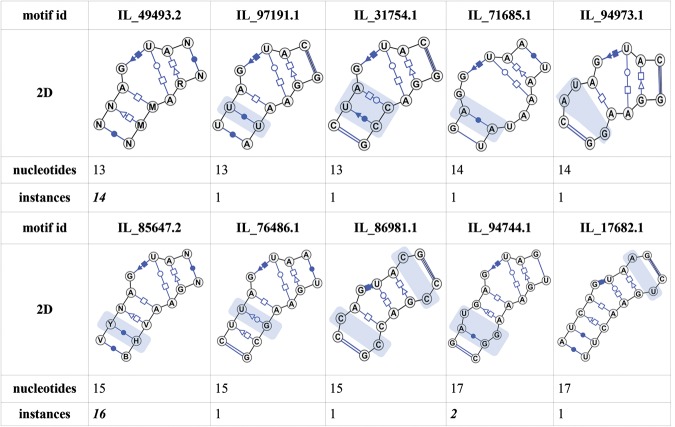
Comparison of 10 motif groups with sarcin–ricin-like features. The 2D diagrams were automatically generated by VARNA (Darty et al. 2009) based on the consensus base-pair signatures of the motif groups. Structural features incompatible with the motif group containing the classic sarcin–ricin motif instances (motif id IL_49493.2) are highlighted with blue overlays.

It is important to note that in automatic classification, certain motif instances will be treated differently than by manual analysis. Automatic classification tends to generate more motif groups according to distinguishing features of each instance, which depends on the quality of the input loop structures. We illustrate this by using the sarcin–ricin motif as an example. Using the facilities for automatic identification of similar motif groups provided in the RNA 3D Motif Atlas, we identified 10 motif groups whose instances include sarcin–ricin and similar motifs (Fig. 5), seven of which are singletons. The three groups with more than one instance are very homogeneous and have very low average intraclusteral geometric discrepancy not exceeding 0.25 Å/nucleotide. The seven singleton groups each possess distinctive characteristics justifying their separation into different groups: Some have additional base pair(s), others have intercalated nucleotides, and others have unique geometries.

Similarly, in the RNA 3D Motif Atlas, there are 15 kink-turn motif groups, all adopting a similar bent shape but differing in the number and type of base pairs or intercalated nucleotides. Flexible motifs, such as the kink-turn, are likely to be separated into different motif groups, because they can adopt a range of conformations. Conversely, highly structured compact RNA 3D motifs are expected to form a small number of conformations. For example, the *C-loop* motif is represented by a single coherent motif group (motif id IL_73276.1).

There are 10 hairpin loop motif groups with *GNRA*-like features. The largest of these (motif id HL_67042.4) includes motif instances with up to two bulged bases, while still having a low average intraclusteral discrepancy of 0.40 Å/nucleotide. Motif instances with sequences not conforming to the GNRA consensus but adopting the same geometry are also successfully identified in motif HL_67042.4 (for example, AGCC or UAAC hairpins). Group HL_67042.4 has roughly four times the number of instances as the other nine groups combined, which tend to have extra stacked bases or different base-pairing interactions.

*T-loop* hairpins are found in two main motif groups: HL_97270.1 with zero or one bulged bases and HL_72498.5 with two or three bulged bases. The bulged bases always occur after the conserved A forming the tWH base pair. The separation of the groups is caused by the criterion shown in Figure 3F. There were also eight more motif groups corresponding to T-loop motif variants.

The *UNCG* hairpin loop motif is represented by a single group with 34 motif instances (motif id HL_39895.3). The core motif contains only 5 nt, but all motif instances have a bulged base in the location corresponding to the “N” from the sequence signature. This base is not part of the core motif because bulged bases are not included in the 3D structural alignments of the motif groups in the Motif Atlas.

### Distribution of motif group size

The distribution of motif instances across motif groups is shown in Table 1. More than one-third of hairpin and internal loop motif groups have only one instance in the NR data set, and two-thirds have five or fewer instances. A relatively small number of motif groups have significant numbers of instances. Only 12 internal loop groups have more than 20 instances, and only two have more than 100 in the NR data set.

**TABLE 1.**
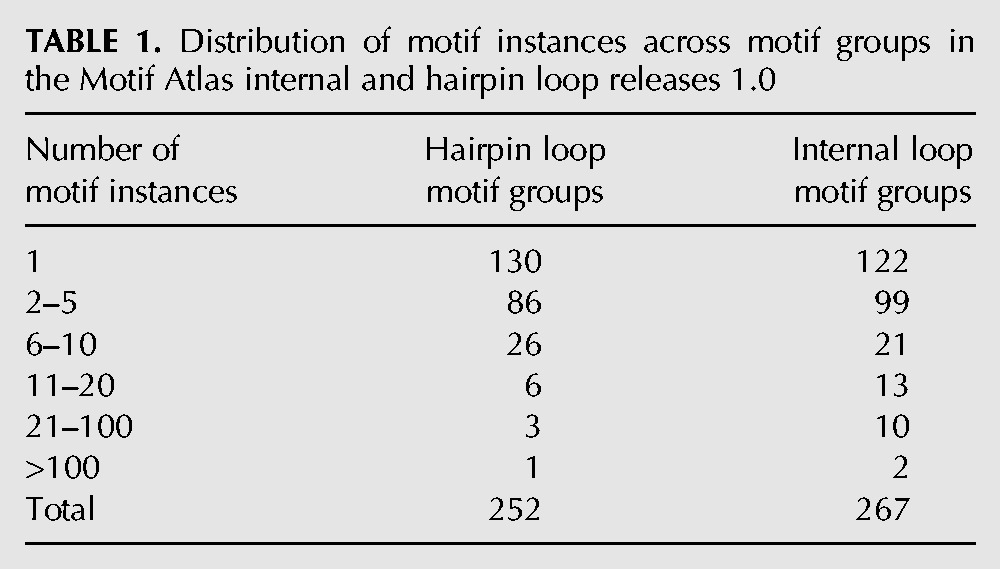
Distribution of motif instances across motif groups in the Motif Atlas internal and hairpin loop releases 1.0

The most populated internal loop motif group represents internal loops having one, two, or three bulged bases occurring in the same chain, in addition to the two flanking canonical base pairs, which in this family, stack on each other (motif id IL_97217.5). This group is closely followed by the 6-nt motif group IL_47174.5, which comprises a noncanonical cWW base pair stacked between two canonical base pairs. Each of these groups is structurally similar to several smaller motif groups.

By far the most populated hairpin loop motif group is the GNRA motif group described above (motif id HL_67042.4). Other hairpin groups with many instances include UNCG tetraloops, T-loop variants, kissing hairpins, tRNA anticodon loops, the MS2 virus RNA Hairpin, and others.

Manual inspection of the singleton motif groups, which only have one instance in the current NR data set, suggests they can be divided roughly into three groups: (1) Some represent motifs that are likely to be rare because they adopt unique geometries, in some cases due to specific interactions with a protein or another RNA fragment (induced fit). (2) Others contain motif instances that appear to be poorly modeled. Similar, better-modeled instances are correctly placed by the algorithm in separate, but related motif groups. With improved modeling, these instances would likely be assimilated into established groups. (3) Finally, some singleton groups appear to be artifacts of the maximum-clique-based clustering algorithm used in the Motif Atlas, which is designed to form the largest possible groups first and put any remaining instances in separate groups, even though they share geometric similarity with other instances.

While the third factor causing the prevalence of singleton motif groups could be addressed by adjusting the clustering algorithm or by selecting a different set of nonredundant motif instances, the first two factors are related to the nature of the input data and are expected to continue having a major impact on RNA 3D motif classification for the foreseeable future. The Motif Atlas highlights the need for more careful modeling of the RNA regions structured by noncanonical interactions and provides an opportunity to study induced fit in RNA by comparing related motif groups, especially those with similar sequences.

### Homology-based classification validation

The 3D structures of ribosomal RNAs are very conserved across organisms separated by hundreds of millions or even billions of years of evolution. Most hairpin and internal loops in rRNA are very conserved in structure if not also in sequence. Therefore, we can use the structurally conserved motif instances from homologous locations in rRNAs of different organisms to assess the success of automatic motif classification.

For example, we find that the motif instances corresponding to the triple-sheared motif from helix h42 of the small ribosomal subunit (SSU) from four different organisms for which the 3D structure of the SSU has been determined are all placed in the same motif group, IL_92321.2, by our algorithm (Table 2).

**TABLE 2.**
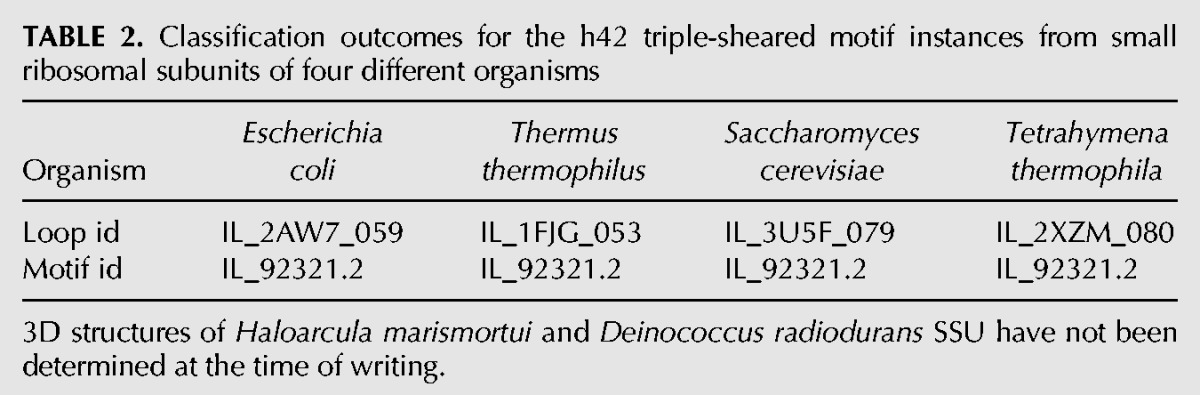
Classification outcomes for the h42 triple-sheared motif instances from small ribosomal subunits of four different organisms

In another example, we compared the classification outcomes of the motif instances corresponding to the kink-turn from H42 of the large ribosomal subunit (LSU) from six different organisms (Table 3).

**TABLE 3.**
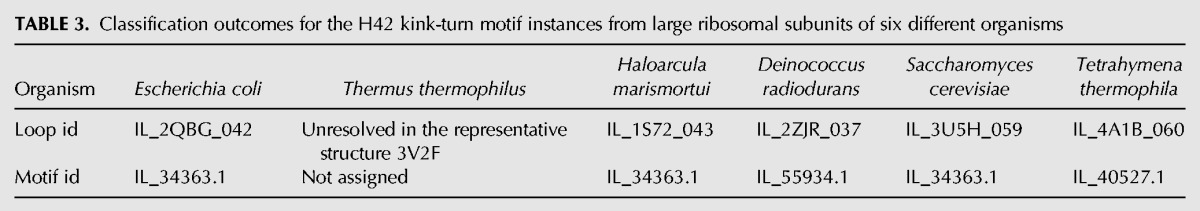
Classification outcomes for the H42 kink-turn motif instances from large ribosomal subunits of six different organisms

Three out of the six motif instances are grouped together in the same motif group IL_34363.1 despite the fact that all three motif instances have different sequences when all bases are considered, and two different sequence variants if only the non-Watson–Crick parts of the motif instances are compared. All motif instances are very similar to each other and have a low average intraclusteral geometric discrepancy of 0.14 Å/nucleotide.

The motif instance from *Deinococcus radiodurans* is placed in a different group, IL_55934.1. This occurs because its flanking base pairs are annotated as “near” cWW rather than “as” cWW. Manual examination indicates that the annotation is correct, but the structure modeling probably is not. As a result, loop IL_2ZJR_037 has two additional nucleotides and is put in a separate motif group. The motif instance from *Tetrahymena thermophila* is a singleton for similar reasons. The closing AU base pair is annotated as “near” cWW, and the motif instance is put in a separate group. Finally, the motif instance from *Thermus thermophilus* H42 is currently not included in the Motif Atlas because helices H42, H43, and H44 are not resolved in the representative structure of the 30S *T. thermophilus* rRNA (PDB 3V2F).

This example illustrates the dependence of classification on the underlying modeling while also highlighting the strength of the established framework, which links together related motif groups. The online resource allows the user to identify related motif groups and find explanations for the clustering decisions. In the future, as FR3D base-pair classification modules are refined and the nonredundant lists evolve, it is possible that all five kink-turn instances will fall in the same motif group.^4^

### Motifs without common names

There are a large number of different internal and hairpin loop motifs, but only a few of these have common names, such as sarcin–ricin or kink-turn, attached to them. For example, a recurrent RNA 3D motif corresponding to the motif group IL_24982.3 has previously been described in the literature (Leontis and Westhof 1998; Petrov et al. 2009), but it does not have a stable, universally recognized name.

Some motifs are currently being referred to by describing their base pairs. For example, the double-sheared or tandem GA motif comprises two stacked trans-Hoogsteen–Sugar, or sheared, base pairs, which are commonly formed by guanines interacting with adenines.

Inspired by this approach, in the RNA 3D Motif Atlas we use the automatically generated “base-pair signatures” for designating motifs without common names. The motif base-pair signatures are constructed by sequentially listing the consensus base pairs, which occur in most motif instances. Under this scheme, the motif IL_24982.3 is labeled as the cWW-tSH-tHW-tHS-cWW motif, and the tandem sheared motif IL_13959.2 is labeled as cWW-tSH-tHS-cWW. Conserved non-base-paired bases are indicated by L or R, depending on which strand they are on as the base pairs are read off. Thus, IL_93424.2, which has a conserved unpaired base on one strand, has base-pair signature cWW-tSH-R-tHW-cWW.

Base-pair signatures capture the main features of internally structured motifs and are consistent with our motif classification methodology. Attaching base-pair signatures is a useful way of creating descriptive and searchable string representations of complex 3D structures. They also make it easier to find common features across several motif groups. For example, most sarcin–ricin motif groups have a base-pair signature ending with -tHH-cSH-tWH-tHS-cWW.

### Analysis of clustering stability across Motif Atlas releases

Three main factors influence the stability of the motif classification process from one release to the next: (1) the dynamics of the 4 Å nonredundant list, which is updated as new structures are released; (2) FR3D structural annotations, which are refined from time to time; and (3) the clustering algorithm, which is also subject to efforts aimed at improvement.

The number of motif groups in the Motif Atlas can change as new 3D structures become available. Generally, this only occurs when a new type of RNA is added to PDB. For new motif instances to be added to the Motif Atlas, the 3D structure where the motif instances are found must either replace the representative structure in the nonredundant equivalence class to which it is assigned or be the founding member of a new equivalence class. The motif instances must also pass all quality-assurance steps described above.

To evaluate the stability of the Motif Atlas with respect to the changes in the nonredundant lists, we retrospectively applied the most current version of the clustering algorithm to seven nonredundant lists separated by 4-wk intervals starting with nonredundant list version 0.85 released on September 1, 2012, and ending with nonredundant list version 1.0 released on March 4, 2013. Seven Motif Atlas releases were produced: 0.7, 0.8, 0.9, 0.10, 0.11, 0.12, and 1.0 (internal loops), and 0.3, 0.4, 0.5, 0.6, 0.7, 0.8, and 1.0 (hairpin loops).

Over this 5-mo period, 133 RNA structure files were added to PDB. Comparison of the nonredundant lists 0.85 and 1.0 reveals that 44 new equivalence classes resulted from these additions, five equivalence classes were represented by a different structure, and nine equivalence classes were updated without changing the representative structure.

Nonetheless, the total number of internal loop groups in the resulting Motif Atlas releases remained stable with 263–267 internal loop groups and 245–252 hairpin loop groups, which suggests that the clustering process is quite stable. The comparison of the motif groups in the September and March releases indicates that most groups did not change. Those groups that did change can be examined in more detail, and their history can be tracked using the online interface. Notably, the versioning and tracking systems implemented in Motif Atlas can be used as a diagnostic tool or to easily put in the current context any study referring to a certain Motif Atlas release.

### Main features of the online resource

Our goal in establishing the RNA 3D Motif Atlas is to create an automated pipeline based on a comprehensive framework for extracting, analyzing, and classifying RNA 3D motifs. All data are presented in a fully transparent and coherent way in the online resource, which is both the final product of the project and the tool for understanding and improving the motif classification and analysis (Fig. 1, Step 14).

#### Display of Motif Atlas releases

Each Motif Atlas release can be viewed either as a list or as a graph. In either view, the users can visualize the motif exemplars in 3D and view the consensus secondary structures generated by VARNA (Darty et al. 2009), which produces the extended secondary structures, including non-Watson–Crick base pairs using the symbols proposed by Leontis and Westhof (2001).

In the list view, users can filter motifs by name or base-pair signature. The list can be dynamically sorted by the number of core nucleotides or by the number of instances in the motif groups. For example, one can quickly identify the largest motif group containing at least one *trans*-Hoogsteen–Sugar base pair or find all sarcin–ricin-like motifs.

The graph view can be used to explore structural similarities of motif groups. The motif groups are represented as nodes connected by edges if at least one instance from one motif matched at least one instance in another. The graph is produced by the Cytoscape Web Browser plug-in (Lopes et al. 2010).

All Motif Atlas releases are archived and stored indefinitely and can also be downloaded for local analysis both manually and programmatically. Any pair of Motif Atlas releases can be compared using a special view accessible from the Motif Atlas homepage, which shows how many motif groups are the same, are present only in one of the two releases, or have been updated. There are many more ways to browse and explore the Motif Atlas, which are explained in the Help section of the site, where users can also find the current documentation regarding the programmatic data access.

#### Display of individual motif groups

**FIGURE 6.**
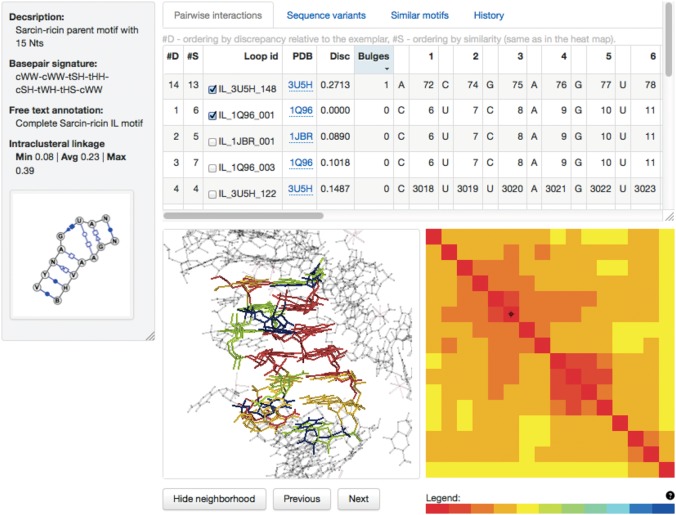
The Motif Atlas web interface for displaying motif groups showing the sarcin–ricin motif group IL_85647.2. The Help pages discuss the components of the interface in detail.

The RNA 3D Motif Atlas has an interactive feature-rich interface for displaying motif groups (Fig. 6), which allows the users to:

- view the secondary structure diagrams produced by VARNA (Darty et al. 2009);
- view the alignment and sequence variability of the instances forming the motif group;
- view base-pair annotations produced by FR3D, tabulated across instances;
- superimpose all instances in 3D and explore their structural context, including RNA, protein, and ligand molecules present in the 16 Å neighborhood;
- identify and explore the differences with similar motif groups;
- track the history of each motif group across all Motif Atlas releases in which it has been present, including its parent and children motif groups; and
- download the motif data.

Individual motif pages automatically list geometrically similar motifs using the results of the all-against-all searches. For example, the sarcin–ricin motif web page (motif id IL_85647.2) provides links to all sarcin–ricin-like motifs depicted in Figure 5. In addition, there are functionalities for interactive comparison of any pair of motif groups, which allow the user to superimpose the motif instances and access the information about structural incompatibilities among the instances of the compared motifs. The reader is referred to the Motif Atlas online help for further details.

## DISCUSSION

### Challenges in motif classification

Previous motif classification methods attempted to organize motifs by their geometry or by their annotated pairwise interactions. However, the geometric measures of discrepancy can be insufficiently sensitive to separate instances of different motifs with similar geometry. Likewise, symbolic pairwise annotations can also be unreliable because most RNA 3D structures are solved to moderate resolution at best, and modeling discrepancies, some of which are likely to be errors, are not uncommon when comparing the same or related structures from independent experimental determinations. The distinguishing feature of our approach is the combination of geometric and symbolic information that feed into the classification algorithm, which is designed to put together instances that can be expected to share the same patterns of sequence variability.

We use symbolic annotations of pairwise interactions only to separate motif instances annotated with different base-pair types (e.g., tHS aligned to cWH) into different motif groups, because different base-pair families have different isostericity rules and thus can be expected to have different patterns of sequence variability (Stombaugh et al. 2009). The instances that remain in a motif group usually have a high degree of agreement among their base-pair annotations, and yet they need not be unanimous. The sarcin–ricin motif group IL_85647.2 is a good example of both phenomena. Nonunanimous base-pair annotations are not uncommon: Of the 145 internal loop motif groups with more than one instance, only 28% have unanimous base-pair classifications at corresponding positions; the other 72% have at least one instance that lacks a base-pair annotation compared with the consensus. Even so, the motif groups are coherent in their overall geometries.

There are many examples of homologous motifs, especially from comparative analyses of the ribosome, that differ structurally only in the presence or absence of bulged bases, which do not change the structure of the core nucleotides or their interactions with other RNA or protein elements. It is therefore desirable to keep these instances together in the same motif group. Our method successfully groups together instances with different locations and numbers (0, 1, 2, …) of bulged bases. Of the 145 internal loop motif groups with more than one instance, only 46% have equal numbers of bulges at all positions. In the other motif groups, there is at least one place where different instances have different numbers of bulged bases, and some motif groups have up to four locations at which this happens. The double sheared motif group IL_13959.2 is a good example; it has very consistent base-pair annotations but has three locations at which the number of bulged bases differs in three instances, all of which come from eukaryotic ribosome structures.

The clustering method is able to group instances correctly, which are not structured by base-pairing as well. For example, the kissing hairpin motif instances (HL_91226.2) are successfully grouped together based purely on their geometry even in the absence of base-pairing interactions within the motif instances. Motif classification algorithms relying solely on base-pair annotations are not expected to perform well with such motifs.

The Motif Atlas correctly categorizes internal loops even when they appear rotated 180° relative to one another in the primary sequence. For example, in the classic sarcin–ricin group (motif id IL_49493.2) nucleotide ranges 165:171 and 138:143 making up the two strands of the motif instance IL_1NBS_007 are aligned with nucleotides 75:81 and 101:106 from IL_1S72_103. Notice how residues with higher nucleotide numbers are aligned with residues with lower nucleotide numbers. These two motif instances also demonstrate that motif geometries and networks of interactions are conserved over nonhomologous positions and are recurrent because IL_1NBS_007 is from RNase P, while IL_1S72_103 is from the 23S rRNA.

### The effect of flipped bases

The geometric discrepancy calculated by FR3D is sensitive to whether a base is modeled in the *anti* or *syn* conformation. Changing from *anti* to *syn* corresponds to a ∼180° rotation about the glycosidic bond, which we call a base flip. “Flipping” one base increases the geometric discrepancy by ∼0.3–0.4 Å/nucleotide for otherwise identical motifs of typical size. Therefore, by using a discrepancy cutoff of 1.0 Å per nucleotide in the geometric comparison step of building the RNA 3D Motif Atlas, we ensure that geometrically similar loops, differing in a single flipped base, are generally identified as matches. In fact, roughly one-third of the motif groups have at least one instance with a base that is flipped relative to the corresponding base in other instances. There is reason to believe that flipped bases in many cases are artifacts of modeling low-resolution data. In fact, many of these cases occur within motif instances from the recently solved eukaryotic ribosome structures, for which homologous motif instances in bacterial ribosomes do not have the flipped bases. Therefore, it seems prudent to keep these motif matches in the same group, rather than splitting them into different groups.

### Annotating motifs in all RNA-containing 3D structures

Using the NR set means that we choose one RNA 3D structure to represent a whole class of structures in the Motif Atlas. Sometimes the representative structure lacks loops that are resolved in other structures. Currently these are not included in the RNA 3D Motif Atlas. An example is the kink-turn from *Thermus thermophilus* H42, which is omitted from the Motif Atlas for this reason (see the section about homology-based validation). To avoid such omissions, it is desirable to annotate motifs in all RNA 3D structures.

The clustering procedure described in this paper yields a representative collection of motif groups, which can be used to annotate all RNA-containing structures in the PDB with RNA 3D motif groups. Our preliminary results suggest the following algorithm for annotating all RNA 3D structures with motif groups from the RNA 3D Motif Atlas: First, a representative instance (centroid) is selected for each motif. Then a modification of the procedure described above is applied, now searching loops from all PDB files using each motif centroid as a query. The structural matches will have to be checked for structural incompatibilities using the same criteria described above. Finally, the loops can be assigned to the motif groups with the lowest geometric discrepancy score. Some loops may not match any centroid with low-enough geometric discrepancy and will be flagged for manual inspection. These may represent new motifs or, more likely, poorly modeled instances of existing motifs. This work will be reported elsewhere.

The classification methods developed here for internal and hairpin loop motifs can, in principle, be extended to three-way and higher-order junctions but will require further development to be implemented successfully, given the greater complexity of these types of motifs.

## CONCLUSION

To summarize, the RNA 3D Motif Atlas is the first regularly updated pipeline for fully automated, comprehensive extraction and classification of internal and hairpin loop motifs from RNA 3D structures. The Motif Atlas has the following key features:

1. The motifs included in the Motif Atlas are taken from a representative, nonredundant set of RNA 3D structures.
2. The resource is automatically updated every 4 wk and is designed to accommodate incremental improvements in methodology and coverage.
3. RNA 3D motifs are compared using both structural annotations and quantitative measures of geometrical similarity.
4. Non-Watson–Crick base-pairing patterns are taken into account in a flexible manner in scoring motif similarity.
5. Variable length insertions are tolerated when these do not affect the core interactions of the motif.
6. All entities are assigned unique and stable identifiers that can be used to track the evolution of the system in time as the database is updated.
7. All Motif Atlas releases are archived and stored indefinitely.
8. The online resource has both human- and computer-friendly interfaces.

## MATERIALS AND METHODS

### Implementation and availability

The RNA 3D Motif Atlas data analysis pipeline is fully automated and runs every 4 wk. The all-against-all structural alignments and geometric discrepancy calculations are carried out using the FR3D software suite (Sarver et al. 2008) and are performed in Matlab under control of Python scripts using Mlabwrap (http://mlabwrap.sourceforge.net/). The data are imported into a MySQL database, which serves as the backend for the Motif Atlas. The website is implemented in PHP and JavaScript. The pipeline uses open-source industry-standard frameworks including CodeIgniter (http://codeigniter.com/), jQuery (http://jquery.com), SQLAlchemy (http://www.sqlalchemy.org/), and Twitter Bootstrap (http://twitter.github.com/bootstrap). In addition, Jmol (http://jmol.org/) is used for 3D structure and Cytoscape Web for graph visualization (Lopes et al. 2010). All software developed as part of this project is available on GitHub at https://github.com/BGSU-RNA.

The Motif Atlas website has been tested on Mac, PC, and Linux computers running a wide range of browsers, including different versions of Chrome, Firefox, Safari, and Internet Explorer. It must be noted that at the time of writing, the Jmol applet is unavailable on some systems due to security concerns caused by using browser plug-ins. The reader is referred to the Motif Atlas online help for further details.

## SUPPLEMENTAL MATERIAL

Supplemental material is available for this article.

## Footnotes

4 In release 1.1, PDB file 2ZJR was replaced by 4IOA as the representative structure for *D. radiodurans* LSU and the corresponding loop instance was placed in group IL_34363.2.
